# A 3D Printed Hydroxyapatite Implant for Temporal Hollowing Reconstruction: A Patient-Specific Approach

**DOI:** 10.3390/cmtr18020028

**Published:** 2025-05-12

**Authors:** Lukas B. Seifert, Alexander Aigner, Sead Abazi, Michel Beyer, Jokin Zubizarreta-Oteiza, Neha Sharma, Florian M. Thieringer

**Affiliations:** 1 Clinic of Oral and Cranio-Maxillofacial Surgery, University Hospital Basel, Spitalstrasse 21, 4031 Basel, Switzerland; 2 Medical Additive Manufacturing Research Group (Swiss MAM), Department of Biomedical Engineering, University of Basel, Hegenheimermattweg 167B/C, 4123 Allschwil, Switzerland

**Keywords:** temporal hollowing, patient-specific implant, virtual surgical planning, hydroxyapatite, maxillofacial surgery

## Abstract

Temporal hollowing, which is a depression in the temple region, often results from trauma, surgical interventions, or neurological conditions. This condition is frequently observed after the resection of encephaloceles, where it can cause esthetic and functional challenges due to temporalis muscle atrophy and nerve palsy. We present a case of a 21-year-old female patient who developed temporal hollowing and complete atrophy of the right temporalis muscle following an encephalocele resection in childhood. The patient also suffered from right-sided frontal nerve branch palsy. To address this complex deformity, a patient-specific implant (PSI) made of hydroxyapatite (HA) was digitally designed and produced using 3D printing technology. The postoperative course was uneventful, with the implant securely positioned and the esthetic result highly satisfactory. This case highlights the potential of 3D printed PSIs in craniofacial reconstruction, offering an optimal solution for both functional restoration and esthetic enhancement. HA further ensures the long-term stability and integration of the implant, providing a promising approach for addressing complex craniofacial defects.

## 1. Introduction

Temporal hollowing, which is a depression of the temple region, is a complex deformity that can have both esthetic and functional impacts on affected patients [1]. This kind of depression often occurs after trauma, surgical interventions, or neurological conditions and is particularly common following the resection of encephaloceles or tumors [1,2]. An encephalocele, a rare congenital brain malformation in which brain tissue protrudes through a skull defect, often requires surgical resection to prevent neurological damage [2]. However, such procedures can result in secondary complications such as muscle atrophy or nerve palsy, leading to long-term esthetic and functional problems [2,3].

Atrophy of the temporalis muscle can also impair masticatory function and jaw movement [1,3]. Reconstruction is challenging because the symmetry and volume of the temple region must be restored. Previous treatment approaches have included autologous tissue transplantation, such as fat or muscle, and alloplastic materials. However, these methods are often associated with complications such as resorption, infection, or unsatisfactory esthetic outcomes [1]. The continuous development of virtual planning and 3D printing technologies has allowed for the production of patient-specific implants (PSIs), which ensure an optimal fit and improve esthetic restoration [4,5,6]. This opened new pathways to precisely reconstruct complex defects like temporal hollowing. In this case, we used these technologies to design a PSI printed in HA. This biocompatible material shares properties similar to natural bone and provides osteoconductive integration potential, enhancing the long-term stability of these implants [4,5,6,7].

## 2. Case Presentation

**Figure 1:**
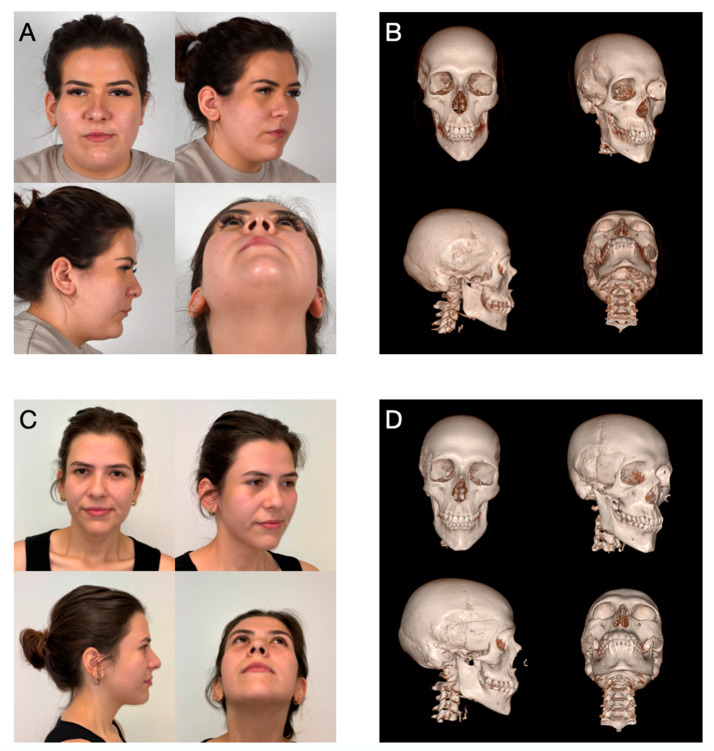
Clinical and computed tomography status before and after the procedure: Preoperatively, both clinically on 24 February 2021 (**A**) and radiologically on 8 March 2024 (**B**), a pronounced deformity in the area of the right temple is evident. Postoperatively, a clinical examination on 15 July 2024 (**C**) shows a homogeneous and symmetrical alignment of the facial contours. The radiological follow-up on 10 July 2024 (**D**) confirms the correct positioning of the PSI.

A 21-year-old female patient presented to our department of oral and maxillofacial surgery with significant temporal hollowing of the right temple region (Figure 1A). This deformity resulted from a prior encephalocele resection in childhood, which led to complete atrophy of the right temporalis muscle. The condition significantly affected the patient both esthetically and psychologically. Additionally, the initial examination revealed a pre-existing right-sided frontal nerve branch palsy that had persisted since the operation in childhood. Comorbidities include migraines and juvenile absence epilepsy, with no seizures since 2020.

### 2.1. Digital Planning

**Figure 2:**
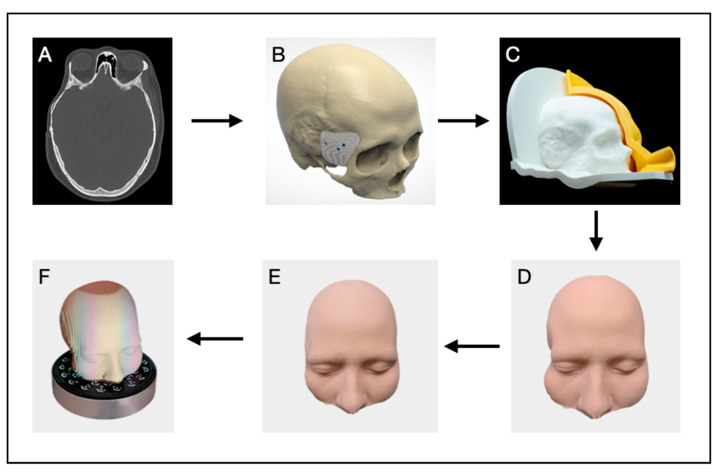
Workflow of the PSI Planning Process: Based on a CT dataset (**A**), a digital PSI plan is created (**B**). A soft tissue facial mask is cast from silicone (**C**). The final facial mask is shown without (**D**) and with the PSI (**E**). The facial mask with the PSI is scanned (**F**).

To achieve optimal esthetic and functional reconstruction, a PSI was designed directly at the point of care (Figure 2). Medical imaging data were acquired using computed tomography (CT) and magnetic resonance imaging (MRI) and were saved in Digital Imaging and Communications in Medicine (DICOM) format (Figure 2A). The datasets were imported into the medical image processing software Materialise Mimics (MIMICS Innovation Suite v. 26.0, Materialise, Leuven, Belgium) to segment hard and soft tissues. A threshold-based segmentation technique was applied to generate 3D models of the skull and the surrounding muscle and skin tissues from the CT and MRI datasets, respectively. The segmented voxel-based 3D models were exported as standard tessellation language (STL) files for further processing.

The implant design was created using the 3D modeling software Geomagic Freeform Plus (v2022.0.34, Oqton Inc., San Francisco, CA, USA) (Figure 2B). A mirrored version of the skull was generated and superimposed onto the original skull model to serve as a reference for reconstructing the geometry of the defect area. Volume correction was estimated through mirroring of the contralateral side and adjusted to compensate for soft tissue atrophy. Approximately 90% volume restoration was targeted. The implant thickness was intentionally increased in the squamous region to compensate for soft tissue thinning resulting from muscle atrophy to approximately 8–10 mm. HA implants are typically not recommended beyond this thickness to maintain mechanical integrity. Multiple design iterations were performed, creating six implant variants, each with an extended surface area over the squamous portion of the temporal bone (Figure 2D).

Both the skull and implant models were imported into Bambu Studio slicing software (BambuLab, V1.10.0.89, Shenzhen, China) and sliced using standard (0.2 mm layer height) and high-quality (0.08 mm layer height) process parameters, respectively. Automatic tree-type supports were added to accommodate overhang regions during printing. All models were manufactured using a fused deposition modeling (FDM) 3D printer (Bambu Lab X1 Carbon, BambuLab, Shenzhen, China) with polylactic acid (PLA) matte (BambuLab, Shenzhen, China) material. Each implant variant was printed in a distinct color for easy identification. Post-processing involved the manual removal of supports and polishing the support–model contact areas with sandpaper of varying grain sizes to ensure a smooth surface finish.

Since an in silico soft tissue simulation was unavailable to predict postoperative outcomes, a silicone skin replica of the patient’s face was created using a molding technique (Figure 2C). The patient’s previously segmented skin model was offset, and a Boolean subtraction was performed in CAD software Geomagic Freeform Plus (v2022.0.34, Oqton Inc., San Francisco, CA, USA) to generate a negative face mold. A two-part mold was designed with integrated venting channels to facilitate air escape and prevent bubble formation during silicone pouring. The molds were fabricated in PLA using the same FDM 3D printer with standard slicing parameters (0.2 mm layer height).

Ecoflex silicone (Smooth-On Inc., Macungie, PA, USA) with a Shore hardness of 00-10 was selected for its skin-like texture and tinted to match the patient’s skin tone. The silicone was poured into the mold, cured for 4 h, and demolded to produce a high-fidelity skin replica. This silicone prototype was assembled with the 3D printed skull model, and the implant variants were sequentially placed in the defect area to assess their impact on the reconstructed skin contour (Figure 2D,E).

To refine the post-operative outcome evaluation, the skull–skin assembly was systematically scanned using a professional-grade structured-light 3D scanner (Transcan C, Shining 3D Tech. Co., Ltd., Hangzhou, China) following the placement of each implant variant (Figure 2F). Then, the digitized models were imported back into the segmentation software and superimposed onto the original patient model for contour analysis. Each resulting skin contour was compared to the digitally mirrored skin model, and the implant that best approximated the ideal contour was selected for final manufacturing and surgical application.

Additional design features were incorporated to facilitate fixation for the chosen implant. Three screw holes were carefully positioned to provide stable attachment. The trajectories of the screws were planned with precision to avoid critical anatomical structures, such as nerves and the ocular globe, ensuring both stability and safety during the surgical procedure. Once the surgeon approved the design, the planning data and the final implant mesh model were sent to CERHUM SA (Liège, Belgium), producing the PSI using stereolithography additive manufacturing (SLA) from HA.

### 2.2. Surgery

**Figure 3:**
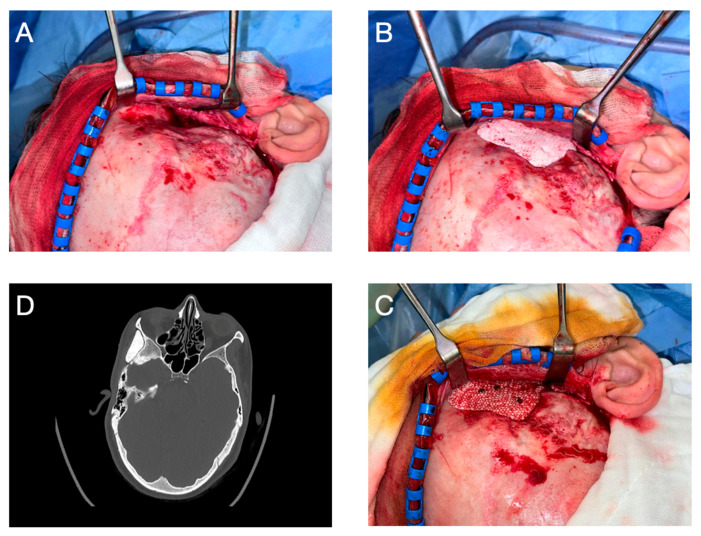
Key surgical steps and postoperative surgical assessment: Exposure of the entire right temporal fossa via a hemicoronal approach (**A**), trial fitting of the PSI (**B**), and fixation of the PSI using three 2.0 osteosynthesis screws (**C**). Correctly positioned PSI in the postoperative CT scan (**D**).

The surgery took place on 19 June 2024 under general anesthesia and antibiotic coverage with co-amoxicillin. Partly utilizing the existing scar from the 2003 resection of a right temporal encephalocele, a hemicoronal incision was made. Following blunt subcutaneous dissection to access the temporal fossa, the temporalis muscle was severely atrophied. To protect the frontal branch of the facial nerve, though palsy was pre-existing, the muscle was dissected in the deep temporal fascia. Protection was routine. After careful exposure of the entire surgical area (Figure 3A), the custom-made PSI from HA, manufactured by CERHUM SA (Liège, Belgium), was tested for fit, which corresponded to the preoperative planning (Figure 3B).

Minor skull irregularities in the area resulting from the childhood neurosurgical procedure were limited so that bone smoothing could be directly performed intraoperatively without preoperative planning. Implant adaptation was confirmed through trial fitting intraoperatively and visually verified against anatomical landmarks and the planned contour. The screw holes were pre-designed during virtual surgical planning. Pilot holes were drilled intraoperatively using a 1.5 mm drill bit and standard techniques aligned with the pre-defined trajectories. The surgical site was then thoroughly irrigated, and the PSI was fixed in place with three 2.0 osteosynthesis screws (Figure 3C). The implant in this case did not come into proximity with the dura or frontal sinus. Finally, a Jackson–Pratt drain was placed with a retroauricular exit on the right side, and the surgical wound was closed in layers.

**Figure 4:**
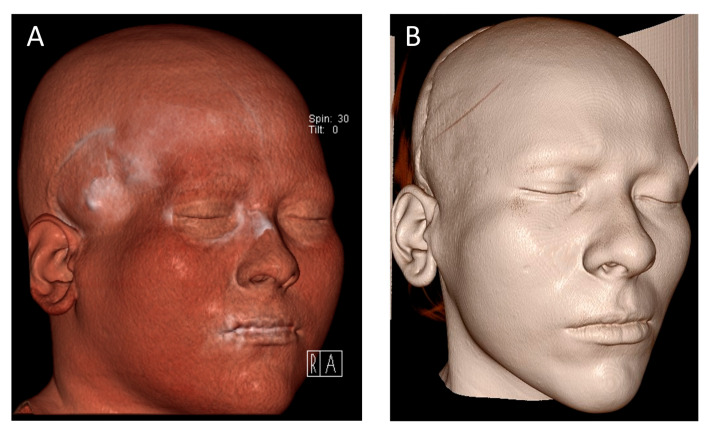
Preoperative 3D surface renderings of the patient’s craniofacial structure. (**A**) Colored volume rendering from a computed tomography (CT) scan displaying pronounced right-sided temporal hollowing, with visible soft tissue atrophy and underlying bony defects. Different lighting as 4B. (**B**) Corresponding grayscale 3D reconstruction illustrating the facial surface morphology in high detail, highlighting the achieved volume augmentation in the temporal region.

**Figure 5:**
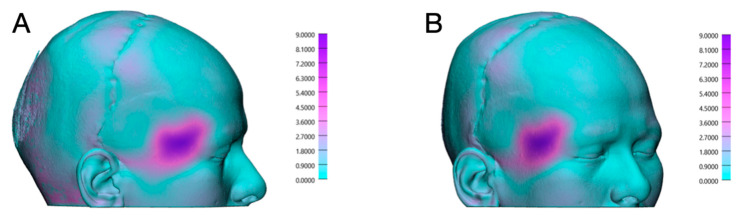
Distance mapping analysis comparing postoperative and preoperative soft tissue structures, with deviations represented in millimeters. (**A**) Right lateral view, illustrating regional discrepancies. (**B**) Antero-lateral right view, providing a detailed perspective on volumetric changes.

After a three-day inpatient stay, the drain was removed, and the patient was discharged for outpatient follow-up care. Postoperative progress, as monitored in regular follow-ups at increasing intervals, was uneventful, allowing for the removal of skin sutures on the tenth postoperative day. Facial sensation and motor function remained intact except for the pre-existing right-sided frontal nerve branch palsy. At submission, clinical follow-up was two months and radiological follow-up was one month. Long-term follow-up is ongoing. The postoperative CT scan on 10 July 2024 confirmed the correct positioning of the PSI (Figure 3D) and the achieved volume augmentation (Figure 4). To precisely assess the differences between preoperative and postoperative CT images, both DICOM datasets were imported into Materialise Mimics, where segmentation of the soft tissue was performed for both time points. The CT images were subsequently superimposed within Mimics to facilitate a direct comparison of the pre- and postoperative soft tissue masks. Further quantitative analysis was conducted in Materialise 3-Matic (v. 18.0, Materialise, Leuven, Belgium) using distance mapping between the segmented soft tissue models. In the region of interest, a volumetric discrepancy exceeding 9 mm was observed, as shown in Figure 5. To evaluate symmetry, a sagittal midplane was generated. The volumetric differences were assessed by comparing the left preoperative segmentation to the right preoperative segmentation and the left postoperative segmentation to the right postoperative segmentation. The results indicated substantial preoperative asymmetry, with a volume difference of 25.04 cm^3^ between the left and right sides. Postoperatively, this difference was significantly reduced to 10.55 cm^3^, demonstrating an improvement in soft tissue symmetry following the intervention.

## 3. Discussion

### 3.1. PSIs in Oral and Maxillofacial Surgery

The use of PSIs for reconstructing functionally and esthetically critical bone structures in the oral, maxillofacial, and craniofacial regions has significantly increased in recent years, and our case adds to the growing body of evidence while demonstrating a specific workflow with HA [4,5,6,7,8,9,10,11,12,13]. This trend is not surprising given the clear advantages and has been extensively discussed in the literature [3,4,5,6,7,8,9,10,11,13]. The precise fit of PSIs allows for stable fixation, promoting faster healing and sparing surrounding structures, particularly those of neurovascular nature [4,6]. Shorter surgical times and lower infection risks improve clinical outcomes, offering a financial balance to the relatively high production costs of PSIs compared to conventional techniques [4,6]. Advances in software have facilitated PSI planning, enabling both external providers and surgeons themselves at the point of care to design the implants, reducing barriers to usage, reducing costs, and increasing availability [3,4,6,7,13]. In the presented case, the resection of the encephalocele was performed prior to the availability of modern planning tools, necessitating a two-stage PSI process. Prospectively, however, a single-stage approach would also be conceivable. Once the decision has been made to use a PSI for reconstruction in the craniofacial region, the question arises regarding the implant’s optimal material.

### 3.2. Choice of Material

The alloplastic materials considered for PSI fabrication with the same workflow as in the presented case can generally be categorized into three main groups as follows: metals, polymers, and ceramics (Table 1) [9]. Titanium is the sole metal currently used in this context due to its excellent biocompatibility and low infection rates. Nevertheless, due to its rigidity, titanium has certain disadvantages as an implant material, such as high costs, imaging artifacts, the impossibility to perform preoperative modifications, and limited protective energy absorption in trauma cases [1,7,9].

Polymethyl methacrylate (PMMA) is a widely used polymer, especially in low-resource settings due to its low production costs, radiolucency, and thermal conductivity. However, PMMA is associated with complications, including infections, fragmentation, and poor biocompatibility [1,7,9,11].

Ceramics, specifically HA, emerged as the ideal material for PSI fabrication in the presented patient case. Due to its structure resembling mineralized bone, HA exhibits excellent biocompatibility and integrates with the surrounding bone over time. This osseointegration reduces the likelihood of implant replacement, a significant advantage for younger patients [9]. Therefore, this material was utilized in the case presented. HA bone grafts have been used for over 40 years in various forms of application, including solid implants and cement pastes, and their long-term stability and safety are well-documented in the literature [4,7,8,14]. The high biocompatibility of HA minimizes the risk of foreign body reactions, and among the three material classes, HA has the lowest infection rates [6,7]. This phenomenon may be attributed to the rapid neovascularization, which enhances immune cell infiltration and facilitates a more robust immune response [6]. As an additional option for temporal volume augmentation, lipofilling represents an effective therapeutic choice, offering an autologous tissue substitute with similar consistency and low complication rates but risk of resorption [1].

Intraoperative modifications of HA PSIs, such as drilling additional screw holes or reshaping, are easily performed, which represents another advantage of the material [4,7]. Additionally, HA shares the same radiopacity as bone, allowing for straightforward postoperative assessments of implant positioning and osseointegration (Figure 1D) [4,6].

The primary disadvantage of HA is its brittle nature, predisposing it to fractures [4,8]. As demonstrated in the presented patient case, this limitation can be mitigated by ensuring sufficient implant size and placement in areas with minimal mechanical stress, such as the temporal fossa. Fractured HA implants may often be left in situ if stability and functionality are maintained due to their osseointegration properties. However, HA is unsuitable for reconstructing large, functional, and biomechanically stressed bones, such as segments of the mandible [4]. Other potential complications associated with HA include the occurrence of wound dehiscence and/or seroma formation following implantation. This is particularly relevant when HA is applied in powder form, such as in implant coatings, or as a cement [12]. In cases of insufficient soft tissue coverage, mechanical tension at the wound site may lead to impaired wound healing. Due to its physical structure, hydroxyapatite may promote fluid accumulation, especially in the absence of rapid cellular infiltration. As a consequence, seroma formation may occur [12,14,15].

## 4. Conclusions

In the present patient case, reconstruction of the bony defect in the temporal bone with a PSI made of HA appears to be an excellent solution. In particular, the high degree of osseointegration associated with this material promises a highly satisfactory outcome even decades after the intervention [4,6,7,8]. The brittleness of HA, its primary disadvantage, is unlikely to be significant in reconstructions without substantial mechanical stress [4,6]. Measures taken to avoid complications included smooth contouring, planned screw positioning, and prophylactic antibiotics. Nonetheless, clinical and radiological follow-up remains essential to monitor the integration of the implant with surrounding structures [4]. This case highlights the possibilities of craniofacial reconstructive surgery in combination with innovative virtual surgical planning and additive manufacturing techniques.

## Figures and Tables

**Table 1:** Overview of alloplastic and autologous materials for temporal hollowing reconstruction.

Material	Advantages	Disadvantages	References
Hydroxyapatite (HA)	Excellent biocompatibility; osseointegration; radiopacity similar to bone; low infection rate.	Brittle; limited use in load-bearing areas; higher cost.	[4,6]
Polyether ether ketone (PEEK)	Good biocompatibility; radiolucent; strong and durable; customizable.	No osseointegration; higher cost; potential for inflammatory response.	[9,10]
Polymethyl methacrylate (PMMA)	Cost-effective; easy intraoperative handling; radiolucent.	High infection risk; no integration with bone; potential fragmentation.	[11]
Titanium	High strength; excellent biocompatibility; suitable for load-bearing applications.	Radiographic artifact; thermal conductivity; non-resorbable; difficult to contour.	[9]
Autologous fat grafting	Biocompatible; natural contour restoration; minimal rejection risk.	Unpredictable resorption; often requires touch-ups.	[1]
Dermal-fat grafts/free flaps	Good tissue match; potentially permanent volume replacement.	Donor site morbidity; longer surgical time; resorption risk.	[1]

## Data Availability

Data are contained within the article.
